# Ancient Exaptation of a CORE-SINE Retroposon into a Highly Conserved Mammalian Neuronal Enhancer of the Proopiomelanocortin Gene

**DOI:** 10.1371/journal.pgen.0030166

**Published:** 2007-10-05

**Authors:** Andrea M Santangelo, Flávio S. J de Souza, Lucía F Franchini, Viviana F Bumaschny, Malcolm J Low, Marcelo Rubinstein

**Affiliations:** 1 Instituto de Investigaciones en Ingeniería Genética y Biología Molecular, Consejo Nacional de Investigaciones Científicas y Técnicas, Buenos Aires, Argentina; 2 Center for the Study of Weight Regulation and Associated Disorders, Portland, Oregon, United States of America; 3 Department of Behavioral Neuroscience, Oregon Health and Science University, Portland, Oregon, United States of America; 4 Departmento de Fisiología, Biología Molecular y Celular, Facultad de Ciencias Exactas y Naturales, Universidad de Buenos Aires, Buenos Aires, Argentina; 5 Centro de Estudios Científicos, Valdivia, Chile; Fred Hutchinson Cancer Research Center, United States of America

## Abstract

The proopiomelanocortin gene (*POMC*) is expressed in the pituitary gland and the ventral hypothalamus of all jawed vertebrates, producing several bioactive peptides that function as peripheral hormones or central neuropeptides, respectively. We have recently determined that mouse and human *POMC* expression in the hypothalamus is conferred by the action of two 5′ distal and unrelated enhancers, nPE1 and nPE2. To investigate the evolutionary origin of the neuronal enhancer nPE2, we searched available vertebrate genome databases and determined that nPE2 is a highly conserved element in placentals, marsupials, and monotremes, whereas it is absent in nonmammalian vertebrates. Following an in silico paleogenomic strategy based on genome-wide searches for paralog sequences, we discovered that opossum and wallaby nPE2 sequences are highly similar to members of the superfamily of CORE-short interspersed nucleotide element (SINE) retroposons, in particular to MAR1 retroposons that are widely present in marsupial genomes. Thus, the neuronal enhancer nPE2 originated from the exaptation of a CORE-SINE retroposon in the lineage leading to mammals and remained under purifying selection in all mammalian orders for the last 170 million years. Expression studies performed in transgenic mice showed that two nonadjacent nPE2 subregions are essential to drive reporter gene expression into POMC hypothalamic neurons, providing the first functional example of an exapted enhancer derived from an ancient CORE-SINE retroposon. In addition, we found that this CORE-SINE family of retroposons is likely to still be active in American and Australian marsupial genomes and that several highly conserved exonic, intronic and intergenic sequences in the human genome originated from the exaptation of CORE-SINE retroposons. Together, our results provide clear evidence of the functional novelties that transposed elements contributed to their host genomes throughout evolution.

## Author Summary

One of the most striking observations derived from the genomic era is the overwhelming contribution of transposed elements to mammalian genomes. For example, 45% of the human genome is derived from mobile element fragments. Although historically viewed as “junk DNA,” transposed elements could also contribute to novel advantageous functional elements in their host genomes, a process called exaptation. Functionally proven examples of exaptation derived from ancient retroposition events are rare. Using an in silico paleogenomic strategy, we unraveled the evolutionary origin of nPE2, a neuronal enhancer of the proopiomelancortin gene that participates in the production of hypothalamic peptides involved in feeding behavior and stress-induced analgesia. We demonstrate that nPE2 originated from the exaptation of a SINE retroposon in the lineage leading to mammals and remained under purifying selection for the last 170 million years. The difficulty in detecting nPE2 origin as an exapted retroposon illustrates the underestimation of this phenomenon and encourages the finding of the many thousands of retroposon-derived functional elements still hidden within the genomes. Their discovery will contribute to a better understanding of the dynamics of gene evolution and, at a larger scale, the origin of macroevolutionary novelties that lead to the appearance of new species, orders, or classes.

## Introduction

The proopiomelanocortin gene (*POMC*) is expressed mainly in pituitary corticotrophs and melanotrophs, as well as in a population of ventral hypothalamic neurons of all jawed vertebrates [1,2]. *POMC* encodes a prohormone that gives rise to several bioactive peptides that include ACTH (adenocorticotropic hormone), α-, β-, and γ-MSH (melanocyte-stimulating hormone) and β-endorphin. A large body of evidence implicates POMC-derived peptides in evolutionarily conserved functions as diverse as the stress response, skin and hair pigmentation, analgesia, and the regulation of food intake and energy balance [3,4].

Pituitary and brain transcriptional regulation of *POMC* are controlled by modular *cis*-acting elements present in the 5′ flanking region [5]. Whereas pituitary-specific *POMC* expression depends on proximal sequences located within 400 bp upstream of the transcription start site, neuronal expression is independently controlled by distal sequences located several kb further upstream [6]. Combining the use of phylogenetic footprinting analysis and transgenic mouse studies, we have recently determined that *POMC* expression in the hypothalamus is conferred by the action of two enhancers, nPE1 and nPE2, located at −12 kb and −10 kb of the mouse *POMC* gene, respectively [5]. Although nPE1 and nPE2 are unrelated at the sequence level, their regulatory functions seem to overlap, since only the removal of both elements from transgenic constructs leads to the loss of reporter gene expression in hypothalamic *POMC* neurons [5].

One of the most compelling observations derived from the recent completion of several genome projects is the overwhelming contribution of transposed elements to mammalian genome composition. For example, 45% of the human genome and 33% of the mouse genome are derived from insertions of transposed elements, the vast majority of which have lost transposable activity [7,8]. Although historically viewed as genome parasites, mobile elements may also participate in gene evolution by providing a large collection of distinct sequences that may contribute to novel functional elements in their host genomes [9,10]. For instance, the number of SINE (short interspersed nucleotide elements) of the primate-specific Alu family in the human genome exceeds one million, and many of them have been reported to function as novel splicing sites, *cis*-regulatory elements, polyadenylation sites, or protein domains [11–13]. A random transposition event may be useful for the host genome if the inserted mobile element directly interacts with neighboring genes or becomes functional after accumulating advantageous mutations. If the novel function improves fitness, the transposon-derived element may become fixed into the genome by purifying selection, a process called exaptation [14,15]. Recently, several high-throughput studies performed in different mammalian genomes detected the presence of thousands of transposed elements that are likely to have been exapted since they are under purifying selection, although their functional properties have not yet been tested [16–20]. Last year, the discovery was reported of several ultraconserved functional sequences in terrestrial vertebrate genomes that originated from ancient exaptation events of SINEs, which were active until recently in the living fossil fish coelacanth [21].

Using an in silico paleogenomic strategy, we demonstrate here that the neuronal *POMC* enhancer nPE2 originated from the exaptation of a CORE-SINE retroposon in the lineage leading to mammals and has remained under purifying selection for the last 170 million years. Functional studies performed in transgenic mice show that two nonadjacent 45-bp regions of nPE2, which are derived from an exapted CORE-SINE retroposon, are essential for enhancer activity in POMC hypothalamic neurons. In addition, we demonstrate the existence of other highly conserved sequences in the human genome that originated from the exaptation of CORE-SINE retroposons. Together, our results provide clear examples of the functional novelties that transposed elements contributed to their host genomes throughout evolution.

## Results

### The *POMC* Enhancer nPE2 Is a Mammalian Novelty

**Figure 1:**
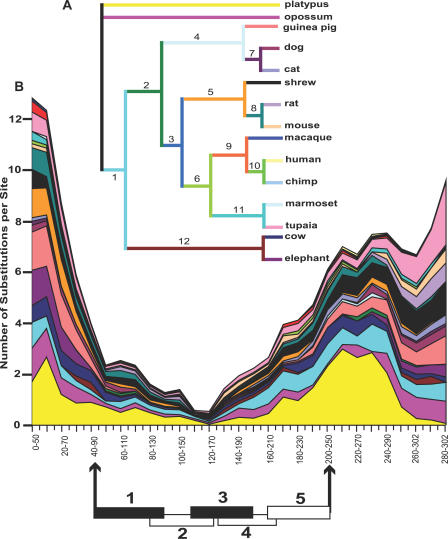
Evolutionary Divergence around Mammalian nPE2 Sequences (A) Phylogenetic relationships among the orthologous nPE2 enhancers of mammals, including monotremes and marsupials. Branches and nodes are displayed with different colors and arbitrary numbers are indicated on branches for quick reference. Note that this tree was built using the phylogenetic relationships among nPE2 sequences and it is not congruent with the phylogenetic trees of the respective species. (B) Evolutionary divergence sliding-window plot of *nPE2*. The histogram shows the number of substitutions per site along each branch of the phylogeny displayed in (A), estimated every ten bases in 50-base intervals. Each layer of the plot represents a branch or node of the tree and colors are maintained with respect to the tree. Substitutions were estimated from the best-fit maximum likelihood model, which incorporates unequal equilibrium nucleotide frequencies and unequal rates of transitional and transversional substitutions (K80). The histograms are stacked so that the total height represents the density of substitutions over the entire phylogeny. Between the two black arrows we indicate the most conserved 160 bp that define nPE2. The five regions into which mouse nPE2 was divided for the deletional analysis are indicated with numbered rectangles. The black regions 1 and 3 were demonstrated to be critical for nPE2 enhancer activity (see Figure 4).

**Figure 2:**
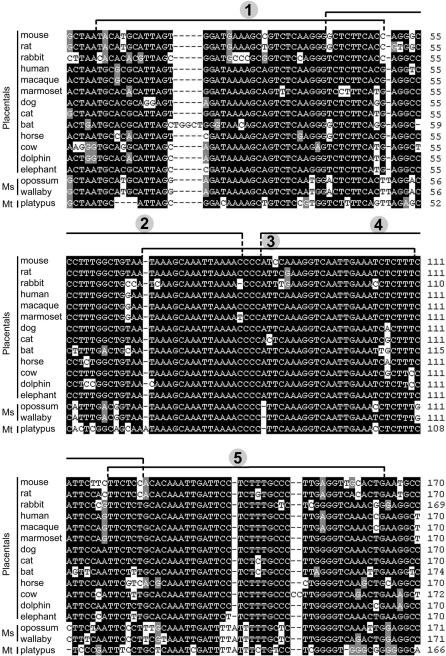
ClustalX Alignment of 16 Mammalian Orthologous nPE2 Sequences Residues highlighted in black and grey indicate different levels of conservation among the orthologs. Mouse nPE2 was subdivided for a deletional analysis into five partially overlapping regions indicated by the brackets and numbered from 1 to 5 within circles. Ms, marsupials, Mt, monotremes.

In previous work, we identified nPE2 enhancers in the genomes of several placental mammals, but not in chicken, the frog Xenopus tropicalis, or in teleost fishes [5]. To trace the evolutionary history of nPE2, we performed further BLAST searches in more recently available mammalian and nonmammalian draft genomes of the Ensembl database and the trace archives of the National Center for Biotechnology Information (NCBI). We retrieved additional ortholog nPE2 sequences from placental mammals (Eutheria) of all available orders (Figures 1 and 2) except Xenarthra (armadillo and sloth), probably due to insufficient sequencing coverage. In addition, we identified nPE2 sequences from the marsupials (Metatheria) short-tailed opossum, and wallaby, as well as from the monotreme (Prototheria) egg-laying platypus. Based on these findings and the absence of nPE2 in nonmammalian vertebrates, we conclude that nPE2 is a mammalian novelty that appeared in an ancestor to all extant mammals, prior to 170 million years ago (MYA) [22].

We next determined the nPE2 regions under stronger selective constraint by performing an evolutionary divergence test that calculates the substitution rate along every branch of a phylogenetic tree constructed with all species analyzed (Figure 1). A sliding-window plot based on the alignment and phylogenetic tree of all ortholog nPE2 mammalian sequences showed an extremely conserved central region within a stretch of approximately 160 bp with substitution rates lower than five nucleotides per site (segment between black arrows in Figure 1). Upstream and downstream of this 160-bp region the aligned sequences are highly divergent, an indication of more relaxed evolutionary constraint (Figure 1). A ClustalX alignment of nPE2 sequences along the most conserved region is shown in Figure 2. The overall conservation of nPE2 sequences among these 16 different mammals is remarkably high, with the most divergent sequences contributed by the nonplacental mammals platypus, wallaby, and opossum (Figure 2). The most striking aspect of this alignment is that the middle part of nPE2 is ultraconserved in Mammalia whereas the 5′ and 3′ extremes appear to be under less strict purifying selection. For instance, near the 5′ part of the enhancer, the sequence ATA/GAAAGC (20–27, numbers refer to the mouse nPE2), which is almost identical in all species including nonplacental mammals, has been changed to ATGCCCGC in the rabbit only. Similarly, the consensus element CATTAG (11–16), which contains a potential recognition site for homeodomain transcription factors, is changed to CAGGAG in the dog only. The number and length of insertions and deletions (indels) in nPE2 across species is quite small. However, some of the indels are phylogenetically informative, such as an A residue in the sequence CCCCATTC (82–90), which is present in all placental nPE2s but missing in basal groups (monotremes and marsupials).

### Deletional Analysis of nPE2 in Transgenic Mice

**Figure 3:**
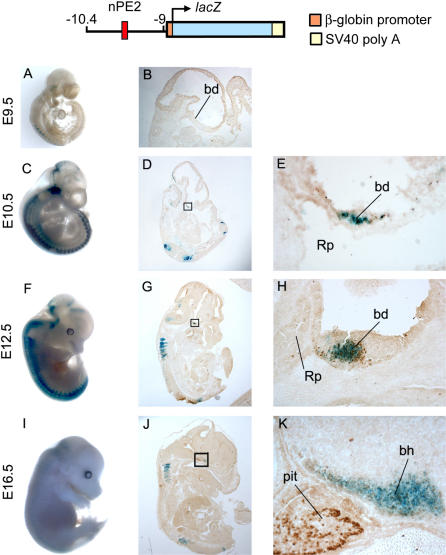
Spatiotemporal *lacZ* Expression Pattern Driven by nPE2 during Embryogenesis A transgene was constructed containing 1.4 kb of mouse *Pomc* upstream sequences including nPE2 (red) ligated to the chicken minimal β-globin promoter (orange) followed by the *E .coli lacZ* reporter gene (blue) and the polyadenylation site from SV40 T antigen (yellow). Embryonic stages (dpc) are indicated on the left. (A, C, F, I) whole-mount *lacZ* staining. (B, D, G, J) *lacZ* staining of sagital sections. The boxes indicate the areas enlarged in the right panels. (E, H, K) magnified views of the diencephalon-pituitary region after combined enzymatic histochemical *lacZ* staining (blue) and anti-ACTH immunostaining (brown). Note that *lacZ* staining is prominent in the basal diencephalon (bd) and later in the basal hypothalamus (bh) but not in Rathke's pouch (Rp) or later in the pituitary (pit). Ectopic expression in somites is present at all developmental stages.

**Figure 4:**
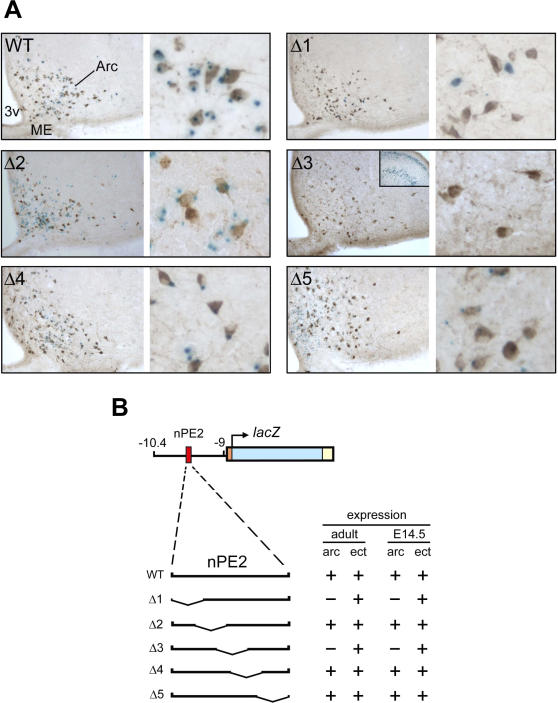
Deletional Analysis of nPE2 Performed in Transgenic Mice (A) Expression analysis of nPE2 deletion constructs in representative animals. Coronal sections at the level of the arcuate nucleus of mice transgenic for each deletion construct, as indicated (WT, Δ1–Δ5). Sections were subjected to *lacZ* staining (blue) followed by anti-ACTH immunostaining (brown) to detect endogenous POMC expression. Left panels show low power views of half an arcuate (10×) and right panels are magnified to show individual POMC neurons (40×). Inset on the left panel of deletion 3 shows ectopic *lacZ* expression in the cerebral cortex of the same brain section, which lacks arcuate expression. For more information see Table S1. (B) Position of the five different deletions (Δ1–Δ5) in relation to WT nPE2 are shown at the left and summaries of *lacZ* expression data obtained for each transgene at embryonic day 14.5 and adulthood are shown at the right. Arc+, coexpression of *lacZ* with ACTH immunopositive POMC neurons in the arcuate nucleus; arc−, absence of coexpression in POMC neurons; ect, ectopic expression of *lacZ*.

A 1.4-kb fragment of the mouse *Pomc* gene containing nPE2, but not nPE1, was ligated upstream of the chicken β-globin minimal promoter followed by the *lacZ* reporter gene and used to generate transgenic mice (Figure 3, top). The ability of nPE2 to drive transgenic expression to POMC neurons was determined by X-Gal staining followed by ACTH immunohistochemistry in coronal brain sections of adult F1 transgenic mice of five independently generated pedigrees (Figures 4A [wild type(WT)] and S1). The reporter gene was eutopically expressed in a high percentage of POMC arcuate neurons in three of the five lines (68%, 61%, and 45%, respectively). Variable patterns of ectopic reporter gene expression were also observed in nonhypothalamic brain neurons of these three transgenic lines (unpublished data). The other two transgenic lines did not express *lacZ* in the brain or other tissues analyzed. To investigate whether the developmental onset of nPE2-driven *lacZ* expression coincides with that of endogenous *Pomc*, we subjected 9.5, 10.5, 12.5, and 14.5 days post coitum (dpc) transgenic embryos to X-Gal staining followed by ACTH immunohistochemistry. Figure 3 shows that both signals are detected in the ventral diencephalon at 10.5 dpc, consistent with the onset of endogenous *Pomc* expression in this brain region [23]. As expected, β-gal activity was not seen in corticotrophs or melanotrophs of the pituitary [5], which start expressing *POMC* at 12.5 and 14.5 dpc, respectively [23]. Thus, reporter gene expression driven by a 1.4-kb genomic fragment containing nPE2 ligated to a heterologous promoter is able to recapitulate the spatial and temporal expression patterns of *Pomc* in mediobasal hypothalamic neurons.

To identify the critical sequences of nPE2 for enhancer activity, we created an additional series of five transgenes carrying discrete overlapping deletions of approximately 45 bp each that we named regions 1 to 5 (Figures 1, 2, and 4). Each deletion was designed by taking into consideration the most conserved segments within nPE2 and the location of potential transcription factor binding sites, as determined using the MatInspector program [24]. For each transgene, we generated multiple transgenic mouse lines and analyzed *lacZ* expression in coronal brain sections from F1 adult animals and sagital sections from F1 or F2 whole embryos at 14.5 dpc. Like the WT construct, transgenes carrying deletions 2, 4, or 5 reliably targeted reporter gene expression to hypothalamic POMC neurons in adults (Figures 4 and S1) and embryos (Figures S2), and the overall expression levels were similar to WT nPE2 transgene. Colocalization of β-galactosidase activity within ACTH immunoreactive hypothalamic neurons was observed in two out of five nPE2-Δ2, three out of five nPE2-Δ4, and eight out of 12 nPE2-Δ5 transgenic mouse lines. In contrast, all five nPE2-Δ1 and six nPE2-Δ3 transgenic pedigrees failed to direct *lacZ* expression to POMC neurons (Figure 4; Table S1). Together, these results indicate that the nPE2 regions encompassing deletions 1 and 3 are essential for enhancer activity, as summarized in Figure 4B. Interestingly, region 3 is the most phylogenetically conserved sequence within nPE2 (Figures 1 and 2) and concentrates the vast majority of potential transcription factor binding sites identified by the MatInspector program (unpublished data).

### In Silico Paleogenomic Identification of nPE2 As a CORE-SINE–Derived Sequence

To investigate the molecular evolutionary process underlying the emergence of nPE2 as a novel mammalian enhancer of *POMC*, we performed BLAST searches for nPE2 paralogs in all available mammalian genomes. In addition to the anticipated 100% hit at its *POMC* locus, the opossum nPE2 sequence matched three short high identity instances on different chromosomes of the opossum genome. These four related sequences were used as queries to reexamine the opossum genome, resulting in additional significant hits that were annotated as SINE-derived sequences in the University of California Santa Cruz (UCSC) Genome Browser. Similar results were obtained from BLAST searches of trace sequences from the wallaby genome.

**Figure 5:**
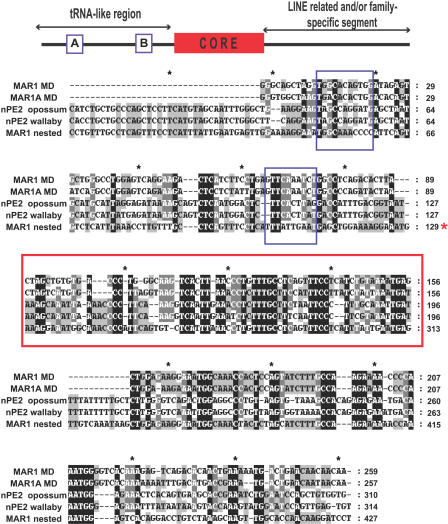
*POMC* Neuronal Enhancer nPE2 Is a CORE-SINE–Derived Sequence Sequence alignment of the opossum and wallaby nPE2 enhancer sequences and three marsupial CORE-SINE consensus sequences from RepBase: MAR1 MD, MAR1a MD, and a representative nested MAR1 located at Chromosome 3 of the opossum genome (according to UCSC Genome Browser). CORE-SINE functional regions are indicated at the top of the schematic. The shading of the alignment is based on the identity of residues and shows percentage of conservation within each column. One hundred percent identical aligned nucleotides are shaded in black. More than 80% conservation is depicted in dark grey shade. Columns with less than 80% and more than 60% conservation are shaded in light grey, whereas nucleotides with less than 60% are not shaded. Sequences corresponding to boxes A and B are depicted in blue boxes, whereas core sequences are within a red box. Black asterisks are presented at 20-nucleotide intervals. The red asterisk indicates the location of the first core sequence of the nested MAR1 that was removed to simplify the figure.

To determine the family of SINE retroposons with greatest similarity to nPE2, we aligned opossum and wallaby nPE2 to representative consensus sequences of the various SINE families obtained from Repbase [25] using ClustalX [26]. Sequence alignments and identity values indicated that nPE2 is most similar to members of the CORE-SINE retroposon superfamily and, particularly, to members of the MAR1 family (Figures 5 and S3; Table 1). CORE-SINEs, V-SINEs, and Deu-SINEs are the three superfamilies of tRNA-like SINEs that were identified among a wide range of species and characterized by a highly conserved central region [18,27–29]. In particular, CORE-SINEs carry a 65-bp “core” sequence and were first described as mammalian-wide interspersed repeats (MIRs) in mammalian genomes [30–32] and later found in nonmammalian vertebrates and invertebrates [27,28]. Sequence comparisons between marsupial nPE2s and consensus MAR1s revealed greatest similarity in the core region, and somewhat less similarity in the 5′ pol III promoter-like region and the 3′ variable region (Figure 5). For example, the identity between opossum nPE2 and MAR1 is 59% along the entire 70 bp of the core and this value rises to 71% in the 45 bp of the core's 3′ end. Table 1 shows the percentage of identity and evolutionary distance between opossum and wallaby nPE2 and core sequences of different CORE-SINEs. Within MAR1s, the values are highest for MAR1a, followed by MAR1 and somewhat lower for MAR1b, which is the most divergent member of this family. Other members of the CORE-SINE superfamily, including Ther1 and Ther2 from placental mammals, Mon1 from platypus, and MIRs from all mammals, display lower levels of identity with nPE2 (Table 1; Figure S3). This sequence divergence explains our initial failure to identify the nPE2 enhancer as a SINE-derived element in nonmarsupial genomes. Interestingly, some MAR1 instances present in the opossum genome have two adjacent cores that probably originated from a duplication event. These particular elements, which we named “nested” MAR1s (Figure S4), are present in marsupial genomes and share an even higher identity with nPE2 at the core level (Figure 5; Table 1). Thus, using this in silico paleogenomic approach, we were able to determine that nPE2 is an evolutionary mammalian novelty derived from the exaptation of a CORE-SINE retroposon.

**Table 1:**
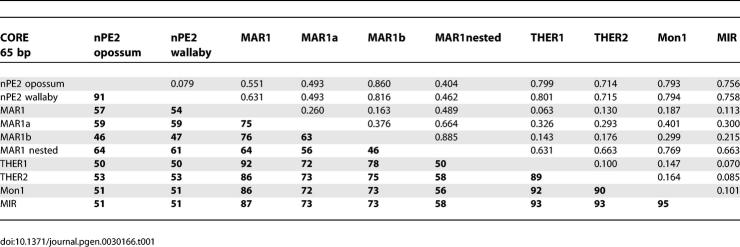
Identity Values (Percent Identical Nucleotides, Bold Numbers) and Evolutionary Distances among Consensus CORE-SINEs and Marsupial nPE2 Sequences

### Are CORE-SINEs Still Active in Marsupials?

**Figure 6:**
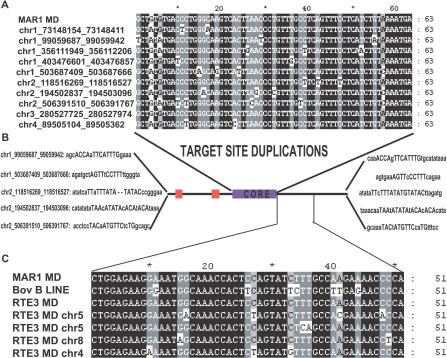
MAR1s Have Been Active Recently in the Opossum Genome (A) Core alignment of the ten highest identity instances of MAR1 in the opossum genome. Chromosome locations are given according to the January 2006 opossum (Monodelphis domestica) draft assembly genome. (B) Selected examples of target site duplications, the chromosome location indicated corresponds to the MAR1 elements shown in part A. Conserved nucleotides at both sides of the duplicated site generated by the retroposon insertion are indicated in capital letters. (C) Alignment of the 3′ end of MAR1, a Bov-B LINE, and RTE3 MD consensus sequences from RepBase and RTE3 instances found in the opossum genome.

Our discovery that the mammalian neuronal *POMC* enhancer nPE2 derives from an ancient CORE-SINE was possible because several MAR1 instances similar to nPE2 are present in marsupial genomes. In fact, we found more than 5,000 recognizable nearly full-length copies of MAR1 in the opossum genome and several hundred of them are low-divergence sequences with identity scores higher than 80%. Table S2 and Figure S5 show the top 20 instances most identical to the consensus MAR1 that are, on average, more than 85% identical to the consensus MAR1 sequence from Repbase. The identity further increased at the level of the core ranging from 89% to 100% (Figure 6A). In addition, we found evidence of target site duplications in many MAR1 instances of the opossum genome, another indication that the retroposition events have occurred recently (Figure 6B). Within the tRNA-like promoter region the conservation is higher in Box B, ranging from 90% to 100%, than in Box A, where the level of conservation is lower in the majority of the instances and much more variable, ranging from 10% to 100%.

CORE-SINEs are nonautonomous retroposons that use the enzymatic machinery of active partner long interspersed nucleotidic elements (LINEs) to create new instances in the genome [33,34]. It has been shown that the interaction between a CORE-SINE and its associated LINE depends on sequence identity between their 3′ ends [27,28]. Therefore, identification of an active MAR1 partner in the opossum genome is an important indication of its current activity. MAR1 mobilization has been suggested to occur through the interaction with the retroposition machinery of members of the Bov-B LINE family [27,28]. We found several Bov-B LINE instances throughout the opossum genome; however, none of them appear to be full-length elements. Interestingly, we found another non-long terminal repeat (LTR) retrotransposon named RTE-3 MD showing 100% conservation along the first 51 bases of the 3′ sequence of the consensus MAR1 (Figure 6C). Bov-B LINEs and RTE-3s belong to the same LINE clade [35] and their consensus sequences are 71% identical. BLAST results indicated that nearly full-length RTE-3 instances are widespread in the opossum genome, which may explain the high copy number of MAR1s detected (Figure 6; Table S2). We obtained very similar results when analyzing trace sequences of the wallaby genome. Altogether, these data indicate that MAR1s are still active mobile elements in marsupial genomes or have been active until very recently. The suggestion that MAR1s and their partner LINE RTE-3 are still active in the opossum genome was also proposed recently [20]. We also found that other members of the CORE-SINE superfamily, such as Ther1 and Ther2, are widely present in the opossum genome. Using consensus sequences of these elements (RepBase) we found approximately 5,000 and 2,500 recognizable copies of Ther1 and Ther2, respectively. Compared to opossum MAR1s, Ther1 and Ther2 displayed lower levels of identity and showed no evidence of recent activity.

**Figure 7:**
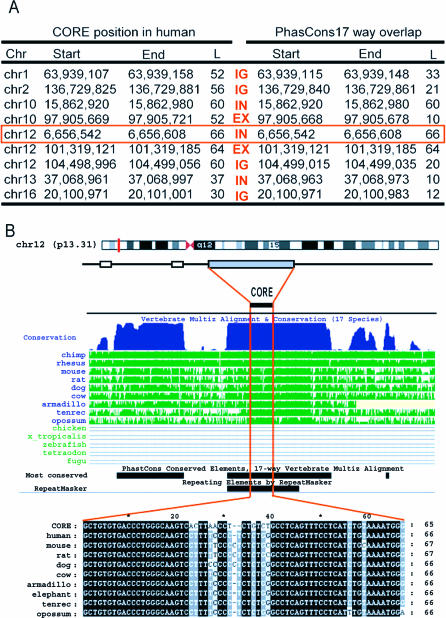
Examples of Additional Exapted CORE SINEs in Mammalian Genomes (A) The locations of nine CORE SINE–derived sequences that overlap with ultraconserved elements predicted by the PhasCons program were determined using the Table Browser at the UCSC Genome Bioinformatics website. The position information of the CORE-SINE–derived sequences and the overlapped ultraconserved elements in human were obtained from genomic sequence data in UCSC Genome Bioinformatics Web site (ver. hg17). Note that these exapted elements are annotated as MIRs in the human genome. The type of sequence is indicated in red in the middle column: IG, intergenic; IN, intronic; EX, exon. The example highlighted within a red rectangle has been detected as an exapted element in [16]. (B) An example of a highly conserved element derived from a CORE SINE located between exons 4 and 5 of ZNF384 at Chromosome 12 in humans (chr12:6,656,542–6,656,608 ). Note that the core-derived sequence is conserved in all mammalian ZNF384 orthologs including opossum.

To our knowledge, nPE2 constitutes the first functionally documented example of a CORE-SINE–derived sequence that was exapted in the mammalian lineage. To investigate whether other phylogenetically conserved instances are derived from CORE-SINE retroposons, we searched for related sequences across orthologous mammalian loci. The MAR1 MD core sequence was compared against the human genome using BLAT (http://genome.ucsc.edu/cgi-bin/hgBlat?command=start) and several thousand similar sequences were detected. Subsequently, we used UCSC Table Browser (http://genome.ucsc.edu/cgi-bin/hgTables) to select the most highly conserved hits across mammals, according to the Most Conserved Elements database (phastCons; [36]). Figure 7A shows the only nine highly conserved hits that we found between the MAR1 MD core and exonic, intronic, or intergenic regions of the human genome. The high conservation of these CORE-SINE–derived sequences in orthologous mammalian loci indicates they have been under strong purifying selection for at least 150 million years. Until experimental proof uncovers their functional role, the presence of these highly conserved CORE-SINE–derived sequences in mammalian genomes will remain a mystery. Figure 7B shows the putative CORE-SINE exaptation event into a highly conserved mammalian sequence present between exons 4 and 5 of the zinc finger transcription factor gene ZNF384/CIZ/NMP4, which is thought to be involved in the regulation of bone metabolism and spermatogenesis [37].

## Discussion

The aim of the present study was to determine the evolutionary history of the *POMC* neuronal enhancer nPE2. We first demonstrated that nPE2 orthologs are highly conserved in their nucleotide sequence in all placental and nonplacental mammals, but absent in other vertebrates. We then performed a systematic search for nPE2 paralogs in all available mammalian genomes and identified three short sequences similar to opossum nPE2 within the opossum genome. The use of these four sequences as queries in further BLAST searches revealed that they are highly similar to various members of the marsupial CORE-SINE retroposon family MAR1. We named the use of progressive searches of genome databases to reconstruct the origin of functional novelties from evolutionary relics “in silico paleogenomics” to distinguish it from the term “paleogenomics,” which is more commonly used in genomic research involving DNA samples obtained from fossil specimens. Our findings are consistent with the hypothesis that an ancient CORE-SINE retroposon was mobilized into the *POMC* locus and exapted as a neuronal enhancer in the lineage leading to mammals more than 170 MYA. Around 30 to 40 million years later, after the split that led to marsupials [22], a group of CORE-SINEs now known as MAR1s started to colonize the marsupial genomes, remaining active until very recently (see Results and Figure 6; also [20,28]). This is in clear contrast to the evolution of CORE-SINEs in placental mammals, which lost transposable activity around 100 MYA and remain now as fossil sequences [28]. The fact that nPE2 is more similar to MAR1s seems to be fortuitous, and suggests that MAR1s are more similar to the ancestral CORE-SINE that was exapted into nPE2 than all other members of the superfamily. The abundance of similar copies of MAR1s within marsupial genomes was key to uncovering the evolutionary origin of nPE2 and indicates that marsupial genomes represent a uniquely positioned source from which to trace the evolutionary origin of mammalian genes. Evidence that nPE2 derives from the exaptation of a CORE-SINE is based on the relatively high percentage of identity between opossum nPE2 and MAR1s (Figure 5). The similarity is especially remarkable in the core region (59%) and even higher along the 45 bp of its 3′ end (71%). This level of identity is comparable to that reported between different MAR1s (MAR1a and MAR1b cores are 63% identical) and to an ancient LF-SINE exapted as a cell-specific enhancer of *ISL1*, which are 61% identical in their most similar region [21]. To our knowledge, the *ISL1* enhancer and nPE2 are the sole functionally proven examples of enhancers whose sequences are derived from ancient retroposons, and nPE2 is the first one discovered to have originated from a member of the CORE-SINE family.

To dissect the regions of nPE2 involved in *POMC* neuronal enhancer function, we performed a deletional analysis in transgenic mice and identified two essential nonadjacent 45-bp sequences: regions 1 and 3. Region 3 is almost absolutely conserved among all species (Figures 1 and 2), suggesting that the array of transcription factors binding to it has probably been constant since the origin of mammals. Interestingly, the 5′ and 3′ halves of region 3 seem to be mutually redundant, since they can be independently removed without impairing reporter gene expression in hypothalamic POMC neurons (deletion of regions 2 or 4). The presence of two A + T-rich motifs (AATTAAAA and AATTGAAA) with potential binding sites for homeodomain transcription factors in each half of region 3 is provocative. In contrast to region 3, the essential region 1 admits many base substitutions, microinsertions, and microdeletions (Figures 1 and 2). However, it is well known that *cis*-acting elements can differ in sequence and still play similar functions, either due to degeneracy in binding site specificity [38] or compensatory mutations in other sites [39]. Region 1 is derived from the 5′ tRNA-like portion of the consensus MAR1, whereas region 3 is derived from the core. This observation is in agreement with other examples of exaptation showing that functionally relevant SINE-derived sequences may come from different portions of the original retroelement [17–19,21]. Based on our findings, it is difficult to know if the CORE-SINE inserted upstream of *POMC* functioned as an enhancer immediately upon its insertion, as proposed for some Alu elements that carry potential binding sites for nuclear receptors [40–42]. Alternatively, the retroposon insertion initially provided adequate raw material for the accumulation of favorable mutations until it evolved into a novel neuronal *POMC* enhancer and became fixed in the lineage leading to mammals, before 170 MYA [22].

Although nPE2 is a mammalian novelty, all jawed vertebrates studied to date, including birds, amphibians, and fishes, express *POMC* in ventral hypothalamic neurons, suggesting that an nPE2-independent regulatory mechanism must control neuronal *POMC* expression in other vertebrates. This is consistent with our recent findings showing that the entire 5′ flanking region of *POMC* from the pufferfish Tetraodon nigroviridis is capable of directing the expression of a reporter gene to POMC pituitary cells but not to POMC hypothalamic neurons of transgenic mice (unpublished data). The ability of nonmammalian vertebrates to express *POMC* in ventral hypothalamic neurons suggests that the appearance of nPE2 probably replaced the function of an earlier *POMC* neuronal enhancer. This puzzle will be resolved when neuronal *POMC* regulatory elements and their cognate *trans*-acting factors from other vertebrates are identified.

Another important conclusion from our study is that exaptation of CORE-SINEs is probably not restricted to nPE2. From several thousand exonic, intronic, and intergenic sequences that we found in the human genome to be derived from the core region of CORE-SINE retroposons, nine of them constitute strongly suggestive examples of exaptation since they are highly conserved among all mammalian ortholog loci. There is a growing list of SINE retroposition events that may have contributed to evolutionary novelties in mammals [9,11,14,43,44], but the vast majority of reported examples correspond to lineage-specific SINEs like Alu and B1 elements present in the primate and rodent genomes, respectively. Since Alu and B1 retrotransposition events are relatively modern, their derived sequences are likely to be easily recognized. However, not all these cases should be considered examples of exaptation until novel adaptive functions followed by purifying selection are confirmed.

More recently, several high-throughput studies detected the presence of transposed element sequences that are likely to have been exapted since they are under purifying selection, although their functional properties have not yet been tested [16–18]. For example, an ancient SINE family that was active in amniotes (mammals, birds, and reptiles) was discovered and named AmnSINE [18]. More than 1,000 AmnSINE-derived instances were found in the human genome and around 10% of them have been under purifying selection in mammals and likely contributed to adaptive novelties in this class. Another recent study demonstrated the existence of thousands of human transposed element fragments under strong purifying selection mostly located near developmental genes [16]. Last year, the discovery was reported of several ultraconserved functional sequences in terrestrial vertebrate genomes that originated from ancient exaptation events of a LF-SINE, which had been active until recently in the living fossil fish coelacanth [21]. Unlike the case of nPE2, recognition of those elements as derived from a LF-SINE was facilitated by the remarkably high level of conservation between the functional tetrapod sequences and the coelacanth retroposon, which must have diverged around 410 MYA.

In summary, our study documents the evolutionary history of a mammalian regulatory element that originated from an ancient retroposition event. The difficulty in detecting the origin of nPE2 as an exapted CORE-SINE retroposon illustrates the underestimation of this phenomenon and encourages the finding of the many more thousands of examples of retroposon-derived functional elements still hidden within the genomes and whose discovery will help us to better understand the dynamics of gene evolution and, at a larger scale, the origin of macroevolutionary novelties that led to the appearance of new species, orders, or classes.

## Materials and Methods

### Sequences and databases.

To find nPE2 ortholog and distant paralog sequences we performed BLAST searches using human or mouse nPE2 sequence as queries against whole-genome assemblies from the Ensembl website (http://www.ensembl.org); we also searched the Trace Archive (http://www.ncbi.nlm.nih.gov/Traces) using megaBLAST [45]. Species used were Mus musculus (mouse), Rattus norvegicus (rat), Cavia porcellus (guinea pig), Oryctolagus cuniculus (rabbit), Homo sapiens (human), Macaca mulatta (macaque), Callithrix jacchus (common marmoset), Tupaia belangeri (tupaia), Canis familiaris (dog), Felis catus (cat), Myotis lucifugus (microbat), Equus caballus (horse), Bos taurus (cow), Tursiops truncatus (bottlenose dolphin), Sorex araneus (shrew), Loxodonta africana (African elephant), Monodelphis domestica (South American short-tailed opossum), *Macropus euge nii* (tammar wallaby), and Ornithorhyncus anatinus (platypus). Trace archives of Dasypus novemcinctus (nine-banded armadillo) and Choloepus hoffmanni (two-toed sloth) were also searched for nPE2 with negative results. nPE2 sequence accuracy was determined by comparing all trace reads spanning the regions and deducing a consensus. Sequences were aligned with ClustalW (http://www.ebi.ac.uk/clustalw) [26]. The alignments were manually refined and edited using GenDoc (http://www.cris.com). Transposed element sequences were obtained at Repbase (http://www.girinst.org). Sequence identity was calculated between aligned pairs of sequences as the number of residues that matched exactly (identical residues). Each sequence was compared to every other sequence. Evolutionary distances were calculated using MEGA version 3.1 [46]. Evolutionary distance between a pair of sequences was measured by the number of nucleotide substitutions occurring between them. We calculated the distance using the Tamura 3-parameter distance model with Rate Uniformity and Pattern Homogeneity.

### Evolutionary divergence.

Sliding windows analysis of substitution rates was performed using HYPHY [47]. We estimated the number of substitution through the best-fit maximum likelihood model K80 (determined by a model test analysis), which corrects for multiple hits, taking into account transitional and transversional substitution rates and differences in substitution rates among sites. Evolutionary rates among sites were modeled using the Gamma distribution and equilibrium nucleotide frequencies were considered to be equal.

### Transgenes.

Transgenes with deletions of nPE2 subregions were made by PCR with megaprimers [48]. Outer primers 1 and 2 amplified a 1.4-kb fragment spanning a region from −10.4 to −9 kb of the 5′ flanking region of mouse *POMC* gene that includes nPE2. Primer 1: 5′-ATACGCGTCGACTAGGCAAGAGATGCCAGCTAGACCTTAC-3′ (SalI site underlined); primer 2: 5′-ATACGGGGTACCTCCAGAAGGCATCCTTGCATAGTGCCTC-3′ (KpnI site underlined). The 1.4-kb amplified fragment was cloned into the SalI and KpnI sites of the pTrap vector [49] to obtain construct WT-nPE2 (Figure 3A). A series of internal primers was designed to perform successive overlapping deletions within nPE2: primer 1a, 5′-CCAAAGGGCCCTTTAGCACAGTAGCCCACC-3′;1b,5′-CTACTGTGCTAAAGGGCCCTTTGGCTGTAA-3′; 2a, 5′-CCTTTGGATGGGCCCTTGAGACGGCTTTCATCCAC-3′; 2b, 5′-CCGTCTCAAGGGCCCATCCAAAGGTCAATTGAAATC-3′; 3a, 5′-AGAAGAAGAATGTTACAGCCAAAGGGCCCTGGTGA-3′; 3b, 5′-CTTTGGCTGTAACATTCTTCTTCTCCACACAAATTGA-3′; 4a, 5′-ATCAATTTGTGTGGGGTTTTAATTTGCTTTATTAC-3′; 4b, 5′-AATTAAAACCCCACACAAATTGATTCCTCTTTGCCCTTGA-3′; 5a, 5′-CTTTATGGCATTGAAGAATGAAAGAGATTTCAATTGA-3′; and 5b, 5′-CTTTCATTCTTCAATGCCATAAAGGGGCCCAAC-3′. Underlined sequences are complementary within each pair of primers and flank each region to be deleted. In the first step, the outer primers were used in combination with the internal primers carrying each deletion (Figure 3B). The following combinations of primers were used: 1/2 (WT nPE2); 1/1a and 1b/2 (Δ1); 1/2a and 2b/2 (Δ2); 1/3a and 3b/2 (Δ3); 1/4a and 4b/2 (Δ4); 1/5a and 5b/2 (Δ5). The PCR fragments produced in the two different sets of reactions performed with primers 1 and 2 were used in a second step as template and megaprimers, which are complementary around the deleted region. A final PCR amplification with outer primers 1 and 2 was performed to generate a −10.4/−9 kb fragment carrying each of the nPE2 deletions. To reduce sequence errors, PCRs were performed with a low number of cycles, high concentration of template, and the turbo Pfu polymerase (Stratagene) in a PTC-200 Peltier Thermal Cycler (MJ Research). Cycling conditions: initial denaturation 94 °C 5 min; ten cycles of 94 °C 5 min, annealing-ramp 60 °C-55 °C 1 min, 72 °C 2 min; ten cycles of 94 °C 2 min, annealing 55 °C 1 min, 72 °C 2 min; final extension at 72 °C 10 min. PCR products were subcloned into pZErOTM-2 (Zero BackgroundTM/Kan Cloning Kit, Invitrogen) and deletions were confirmed by sequencing before cloning of inserts into the SalI and KpnI sites of pTrap.

### Transgenic mice production.

Prior to microinjection, all transgenes were digested with NotI, eluted from an agarose gel, and purified with the Elutip-D system (Schleicher & Schuell). After precipitating with 3 M sodium acetate (pH 5.2) and 100% ethanol, the DNA was washed with 70% ethanol and resuspended with microinjection buffer (5 mM Tris-HCl, pH 7.4; 0.1 mM EDTA). Transgenic mice were generated by pronuclear microinjection of B6CBF2 zygotes as described previously [6]. Microinjected zygotes were transferred to the oviduct of B6CB pseudopregnant females. Transgenic pups were identified by tail genomic DNA PCR with the following primers: LPZ (5′-TCCCAGTCACGACGTTGTAAAACG-3′) and P (5′-GGTACCGCATGCGATATCGAGCTC-3′), which amplify a transgenic-specific 166-bp fragment. The deletions were detected with primers flanking the element nPE2: delta 2.5 (5′-TGATTTTACTTGGGCCTC-3′) and delta 2.3 (5′-TCAGGCTTGTTCCCATCC-3′) that amplify 340-bp fragments from the endogenous gene and 300-bp fragments from the transgenes with the deletions, respectively. Animals were kept in a ventilated rack (Thoren Caging Systems) under a 12-h light/dark cycle and 20–22 °C room temperature (RT). All procedures using live animals were approved by the respective Institutional Animal Care and Use Committees and followed the Public Health Service guidelines for the humane care and use of experimental animals.

### X-Gal staining and immunohistochemistry.

Transgene expression was determined in F1 adult mice of each independently generated pedigree. Mice were deeply anesthetized, perfused with 4% paraformaldehyde (PFA) in KPBS (0.9% NaCl, 16 mM K_2_HPO_4_, 3.6 mM KH_2_PO_4_, pH 7.4), and brains were excised, postfixed in 4% PFA-KPBS 1 h 20 min at 4 °C, and sectioned (50 μm) in a Vibratome 1000 (Ted Pella). Brain slices were stained with 1 mg of 5-bromo-4-chloro-3-indolyl-β-D-glucuronic acid (X-Gal)/ml in staining solution (PBS [pH 7.3] containing 2.12 mg of potassium ferrocyanide/ml, 1.64 mg of potassium ferricyanide/ml, 2 mM MgCl_2_, 0.01% sodium deoxycolate, and 0.02% NPO-40) for 4 h at 37 °C. After X-Gal staining, brain slices were treated with 1% H_2_O_2_ in KPBS for 40 min, washed twice with KPBS, and incubated overnight at 4 °C with a rabbit polyclonal anti-ACTH-IC-1 (National Hormone and Peptide Program, Harbor-UCLA Medical Center Research and Education Institute, Torrance, California) diluted 1:1,000 in KPBS-0.3 % Triton X-100 and 2% normal goat serum. The next day slices were washed in KPBS and incubated with biotinylated anti-rabbit immunoglobulin G antibody (Vector) diluted 1:200 in KPBS-0.3 % Triton X-100 for 2 h at RT. After washing in KPBS, slices were incubated with avidin/biotin-horseradish peroxidase complex (Vectastain Elite ABC kit, Vector) for 1 h at RT, washed in KPBS, and developed with 2.5% of diaminobenzidine (DAB, Sigma) and 0.05% H_2_O_2_ in TBS (150 mM NaCl, 50 mM Tris-HCl, pH 7.5). Stained slices were then mounted onto 1% gelatin-coated slides (in 0.1% KCr(SO_4_)_2_). X-Gal/ACTH analysis was performed in at least ten different sections per hypothalamus of at least two different independent lines carrying the same transgene. Generally, two transgenic siblings per pedigree were analyzed (see other details in Table S1).

Developmental studies for each transgene were performed in timed pregnant dams obtained by mating B6CBF1 stud males with F0 or F1 transgenic females from a representative transgenic pedigree. After killing the pregnant dam at defined dpc, embryos were removed immediately, washed with KPBS, and fixed with 4% PFA-KPBS for 20 min (E9.5–E12.5) or 30 min (E14.5–E16.5). Fixed embryos were stained whole-mount with X-Gal (37 °C for 4 h), dehydrated with sucrose 30% in KPBS at 4 °C overnight and, the next day, sliced in a cryostat (20 μm). Sections were air dried at RT overnight and postfixed with cold 4% PFA 10 min and washed with KPBS. X-Gal staining was performed at 37 °C for 4 h, followed by anti-ACTH immunohistochemistry as described above, with some modifications. Briefly, the slides were incubated in 1% H_2_O_2_-KPBS for 30 min at RT with light shaking, washed twice with KPBS, and incubated with anti-ACTH (1:300) antibody for 4 h at 37 °C. After washing with KPBS, slices were incubated with secondary antibody (1:200) 1 h at 37 °C, washed with KPBS, and incubated with the Vectastain Elite ABC kit (Vector) or 1 h at RT. Finally, slices were developed with DAB.

### Conservation of CORE-SINE sequences among mammals.

To find more examples of exapted CORE-SINE sequences, we used BLAT to search the Core sequence of MAR1 MD depicted in Figure 6 against the human genome assembly (hg18) at http://genome.ucsc.edu. From the obtained output we selected only those hits that overlapped with the Most Conserved (phastConsElements17way) Track using the Table Browser at http://genome.ucsc.edu/cgi-bin/hgTables. This track contains predictions of conserved elements that were obtained by running phastCons [36] on the multiple alignments generated using multiz on best-in-genome pairwise alignments generated for each species using BLASTZ, followed by chaining and netting.

## Supporting Information

Figure S1 Transgenic Mouse Analysis of nPE2 Deletion Constructs Additional transgenic mouse lines from those shown in Figure 4. Vibratome sections of transgenic mouse brains at the level of the arcuate nucleus of the hypothalamus were subjected to X-Gal staining (blue) followed by anti-ACTH immunohistochemistry (brown). Panels show 10× (left) and 40× (right) objective magnifications. Brains were cut coronally except for lines Δ2–66, Δ5–23, Δ5–25 and Δ5–52, which were cut sagitally. Arrowheads indicate neurons in which ACTH and X-Gal colocalize. Line Δ1–11 illustrates a case in which no X-Gal staining was observed in transgenic hypothalami. See Table S1 for the total number of transgenic lines obtained for each construct. Transgenic mouse line numbers are indicated by the deletion followed by the number of the F0. 3v, third ventricle.
(4.7 MB PDF)

Figure S2 Expression of nPE2 Deletion Constructs in Transgenic Embryos Embryos at 14.5 dpc were sliced in a cryostat, stained with X-Gal (blue), and subjected to anti-ACTH immunohistochemistry (brown). Transgenic lines shown here are the same as those in Fig 4. Colocalization of ACTH /X-gal is clearly seen in all embryos except in Δ1 and Δ3 constructs. Some ACTH cells in Δ1 seem to stain for X-Gal but analysis of adult mice does not support colocalization. Analyses of Δ1-nPE2 adult animals do not show any indication of colocalization (Figures 4 and S1). Inset in the left panel of Δ3–31 shows ectopic X-Gal expression in somites. *POMC* cells of the pituitary are negative for X-Gal in all transgenic lines. Pit, pituitary; hyp, ventral hypothalamus.
(6.0 MB PDF)

Figure S3 *POMC* Neuronal Enhancer nPE2 Is a CORE-SINE–Derived Sequence Most Similar to MAR1s Sequence alignment of the opossum and wallaby nPE2 enhancer sequences and the core region of seven CORE-SINE consensus sequences from RepBase: MAR1 MD, MAR1a MD, MAR1b MD, Ther1, Ther2, MIR, Mon-1, and a representative nested MAR1 located at chromosome 3:509568325–509568397 of the opossum genome (according to UCSC Genome Browser). CORE-SINE functional regions are indicated at the top of the schematic. The shading of the alignment is based on the identity of residues and shows percentage of conservation within each column. One hundred percent identical aligned nucleotides are shaded in black. More than 80% conservation is depicted in dark grey shade. Columns with less than 80% and more than 60% conservation are shaded in light grey, whereas nucleotides with less than 60% are not shaded. Asterisks are presented at ten-nucleotide intervals.
(217 KB PDF)

Figure S4 Nested MAR1s at the Opossum Genome Sequence alignment of 18 instances of nested MAR1s at the opossum genome (A and C; positions in the genome are given according to the UCSC Genome Browser). In (B), a schematic representation of the structure that is more frequently found at the genome as a consequence of the insertion of these elements. Only regions corresponding to the cores are shown in the alignment. The first sequence in the alignment corresponds to two core consensus sequences that were pasted together and used as queries in order to identify these elements through BLAT and BLAST searches. The shading of the alignment is based on the identity of residues and shows percentage of conservation within each column. One hundred percent identical aligned nucleotides are shaded in black. More than 80% conservation is depicted in dark grey shade. Columns with less than 80% and more than 60% conservation are shaded in light grey, whereas nucleotides with less than 60% are not shaded. Asterisks are presented at ten-nucleotide intervals, ∼ symbols are used to indicate the absence of a sequence of 50 nucleotides that is not shown in the figure.
(319 KB PDF)

Figure S5 Top 20 Instances Most Identical to the Consensus MAR1 The shading of the alignment is based on the identity of residues and shows percentage of conservation within each column. One hundred percent identical aligned nucleotides are shaded in black. More than 80% conservation is depicted in dark grey shade. Columns with less than 80% and more than 60% conservation are shaded in light grey, whereas nucleotides with less than 60% are not shaded. Asterisks are presented at ten-nucleotide intervals. Positions at the genome are given according to the UCSC Genome Browser.
(359 KB PDF)

Table S1 Summary of the Expression Analysis Performed by X-Gal/ACTH Double Histochemistry on Adult Brains at the Arcuate Level of WT nPE2 and the Five Deletion Constructs (31 KB DOC)

Table S2 Identity Values (Percent Identical Nucleotides) among Consensus MAR1 MD and the Top 20 Most Similar Sequences Retrieved after BLAST Searches of the Monodelphis domestica Genome Genomic location of the instances is given according to the Genome Browser at UCSC.
(42 KB DOC)

## Abbreviations

ACTH: adenocorticotropic hormone
dpc: days post coitum
LINE: long interspersed nucleotide element
MIR: mammalian-wide interspersed repeat
MYA: million years ago
POMC: proopiomelanocortin
RT: room temperature
SINE: short interspersed nucleotide element
WT: wild type
