# Supplementary material for: Ancient Exaptation of a CORE-SINE Retroposon into a Highly Conserved Mammalian Neuronal Enhancer of the Proopiomelanocortin Gene
Source: PLoS Genet. 2007 Oct 5;3(10):e166. doi: 10.1371/journal.pgen.0030166 (PMC2000970; doi:10.1371/journal.pgen.0030166)
Supplement: Supplementary file 1 [file pgen.0030166.sg001.pdf]

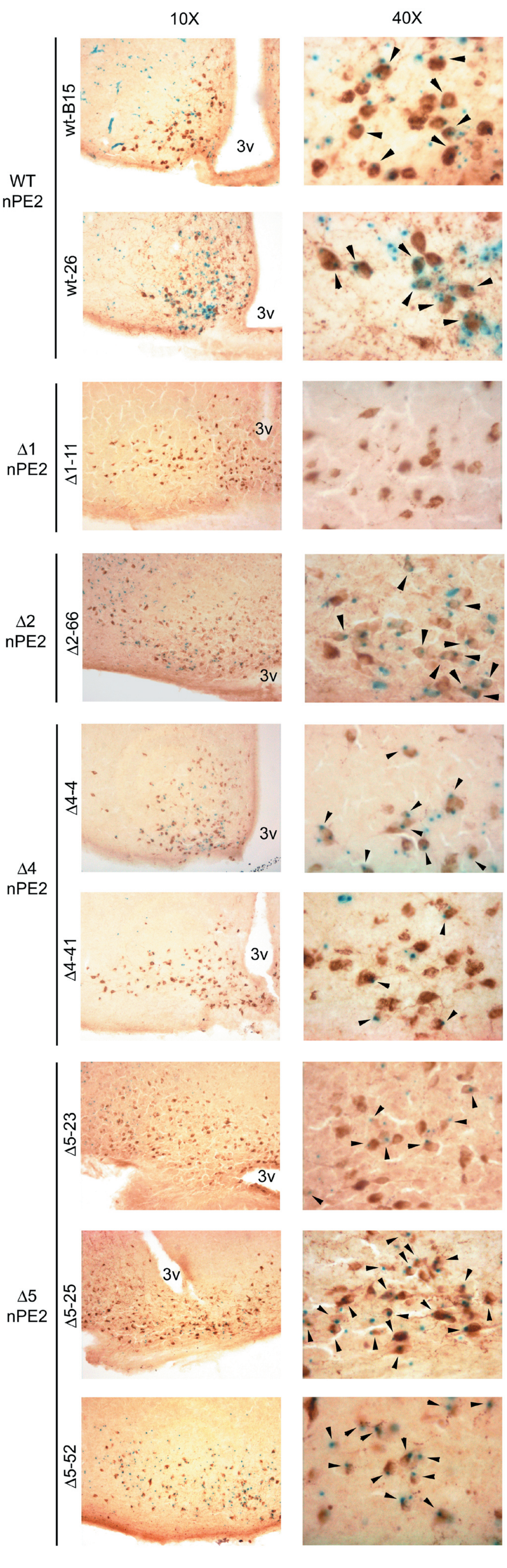

10X
40X
WT nPE2
wt-B15
wt-26
3v
Δ1 nPE2
Δ1-11
Δ2 nPE2
Δ2-66
Δ4 nPE2
Δ4-4
Δ4-41
Δ5 nPE2
Δ5-23
Δ5-25
Δ5-52
