# Supplementary material for: Ancient Exaptation of a CORE-SINE Retroposon into a Highly Conserved Mammalian Neuronal Enhancer of the Proopiomelanocortin Gene
Source: PLoS Genet. 2007 Oct 5;3(10):e166. doi: 10.1371/journal.pgen.0030166 (PMC2000970; doi:10.1371/journal.pgen.0030166)
Supplement: Supplementary file 2 [file pgen.0030166.sg002.pdf]

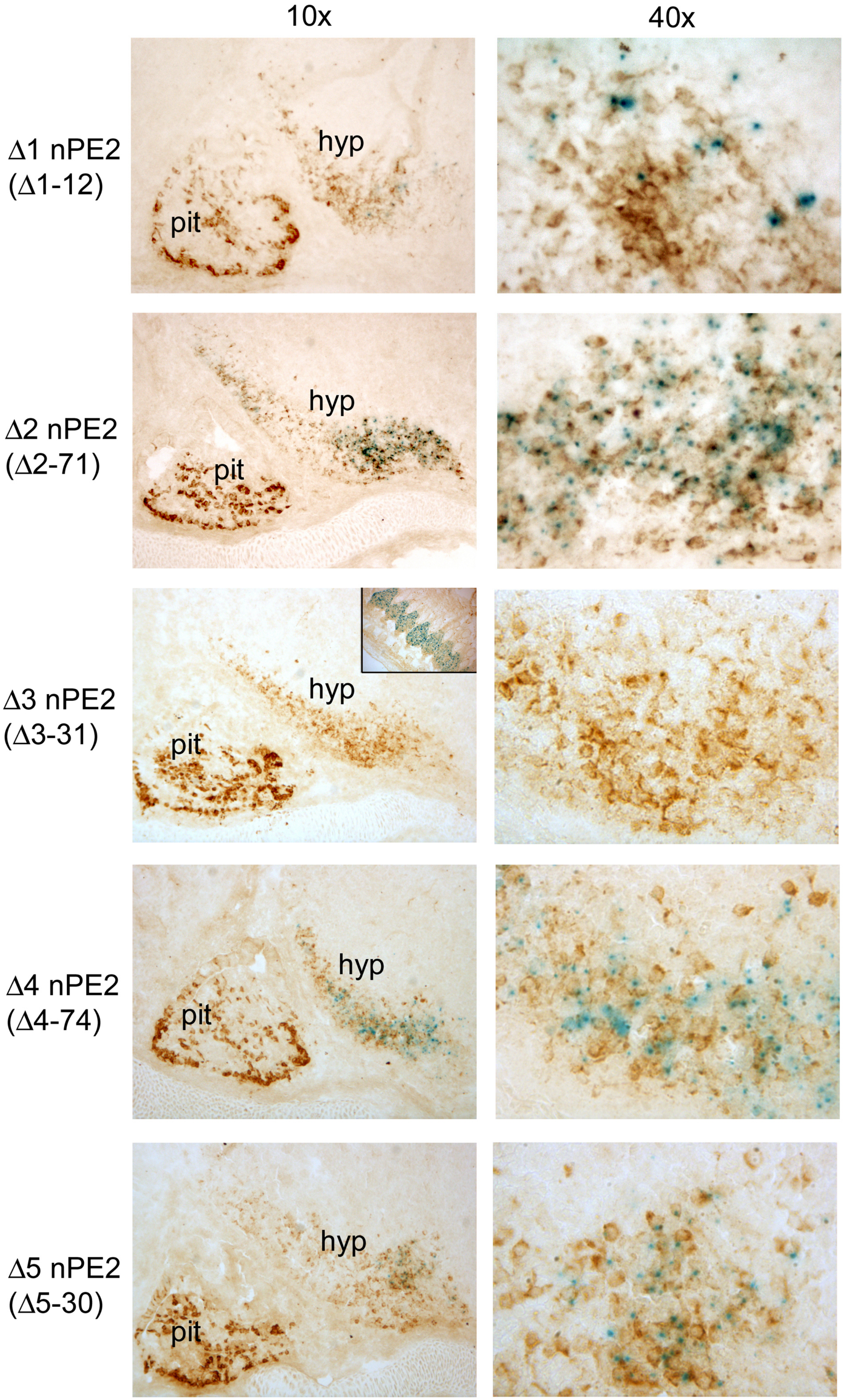
10x
40x
Δ1 nPE2
(Δ1-12)
hyp
pit
Δ2 nPE2
(Δ2-71)
hyp
pit
Δ3 nPE2
(Δ3-31)
hyp
pit
Δ4 nPE2
(Δ4-74)
hyp
pit
Δ5 nPE2
(Δ5-30)
hyp
pit
