# Supplementary material for: Ancient Exaptation of a CORE-SINE Retroposon into a Highly Conserved Mammalian Neuronal Enhancer of the Proopiomelanocortin Gene
Source: PLoS Genet. 2007 Oct 5;3(10):e166. doi: 10.1371/journal.pgen.0030166 (PMC2000970; doi:10.1371/journal.pgen.0030166)
Supplement: Supplementary file 3 [file pgen.0030166.sg003.pdf]

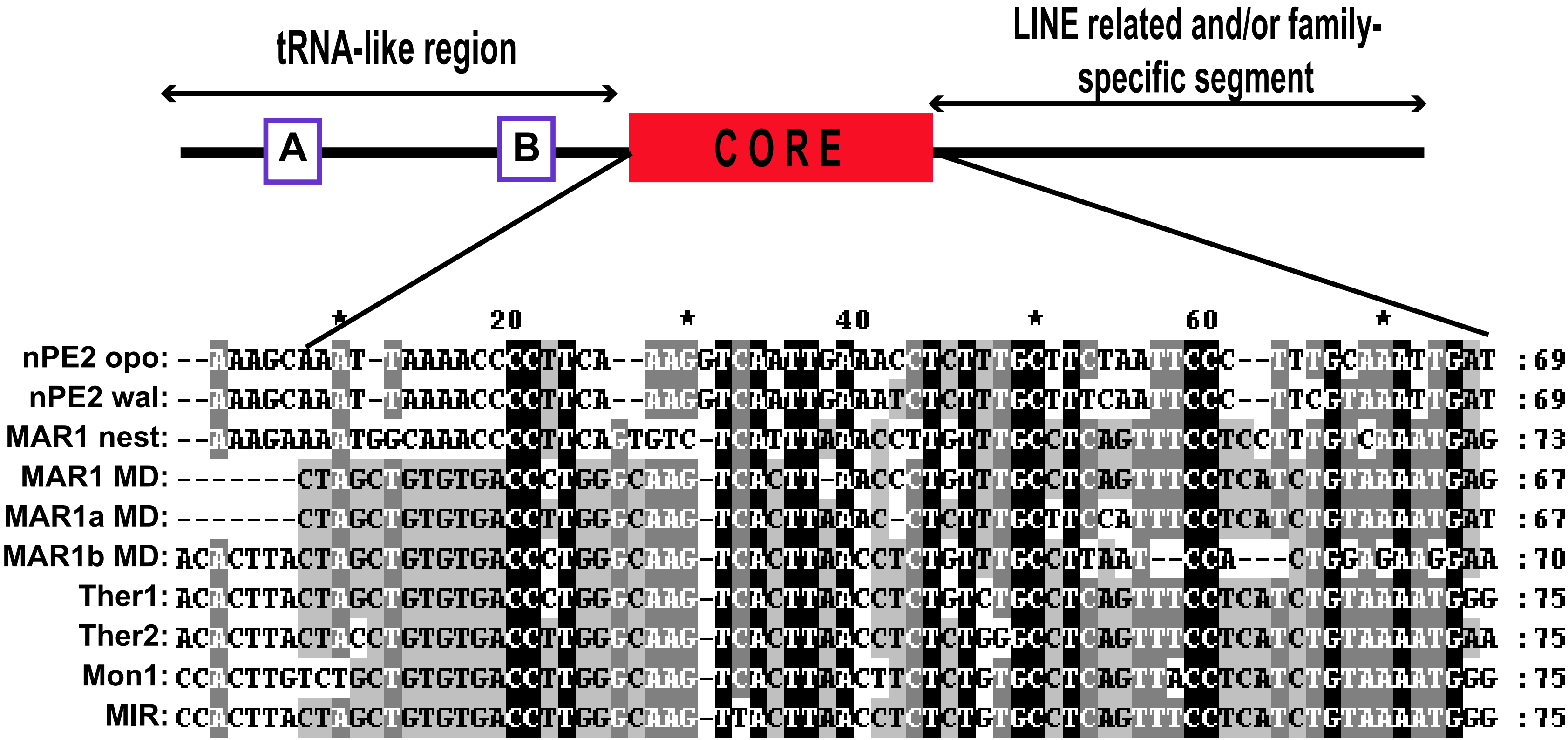
tRNA-like region
LINE related and/or family-specific segment
A
B
CORE
* 20 * 40 * 60 *
nPE2 opo: --AAAGCAAAT-TAAAACCCCTTCA--AAGGTCAATTGAAACCTCTTTGCTTCTAATTCCC--TTTGCAAATTGAT : 69
nPE2 wal: --AAAGCAAAT-TAAAACCCCTTCA--AAGGTCAATTGAAATCTCTTTGCTTTCAATTCCC--TTCGTAAATTGAT : 69
MAR1 nest: --AAAGAAAATGGCAAACCCCTTCAGTGTC-TCATTTAAACCTTGTTTGCCTCAGTTTCCTCCTTTGTCAAATGAG : 73
MAR1 MD: --------CTAGCTGTGTGACCCTGGGCAAG-TCACTT-AACCCTGTTTGCCTCAGTTTCCTCATCTGTAAAATGAG : 67
MAR1a MD: --------CTAGCTGTGTGACCTTGGGCAAG-TCACTTAAAC-CTCTTTGCTTCCATTTCCTCATCTGTAAAATGAT : 67
MAR1b MD: ACACTTACTAGCTGTGTGACCCTGGGCAAG-TCACTTAACCTCTGTTTGCCTTAAT--CCA---CTGGAGAAGGAA : 70
Ther1: ACACTTACTAGCTGTGTGACCCTGGGCAAG-TCACTTAACCTCTGTCTGCCTCAGTTTCCTCATCTGTAAAATGGG : 75
Ther2: ACACTTACTACCTGTGTGACCTTGGGCAAG-TCACTTAACCTCTCTGGGCCTCAGTTTCCTCATCTGTAAAATGAA : 75
Mon1: CCACTTGTCTGCTGTGTGACCTTGGGCAAG-TCACTTAACTTCTCTGTGCCTCAGTTACCTCATCTGTAAAATGGG : 75
MIR: CCACTTACTAGCTGTGTGACCTTGGGCAAG-TTACTTAACCTCTCTGTGCCTCAGTTTCCTCATCTGTAAAATGGG : 75
