# Supplementary material for: Ancient Exaptation of a CORE-SINE Retroposon into a Highly Conserved Mammalian Neuronal Enhancer of the Proopiomelanocortin Gene
Source: PLoS Genet. 2007 Oct 5;3(10):e166. doi: 10.1371/journal.pgen.0030166 (PMC2000970; doi:10.1371/journal.pgen.0030166)
Supplement: Supplementary file 4 [file pgen.0030166.sg004.pdf]

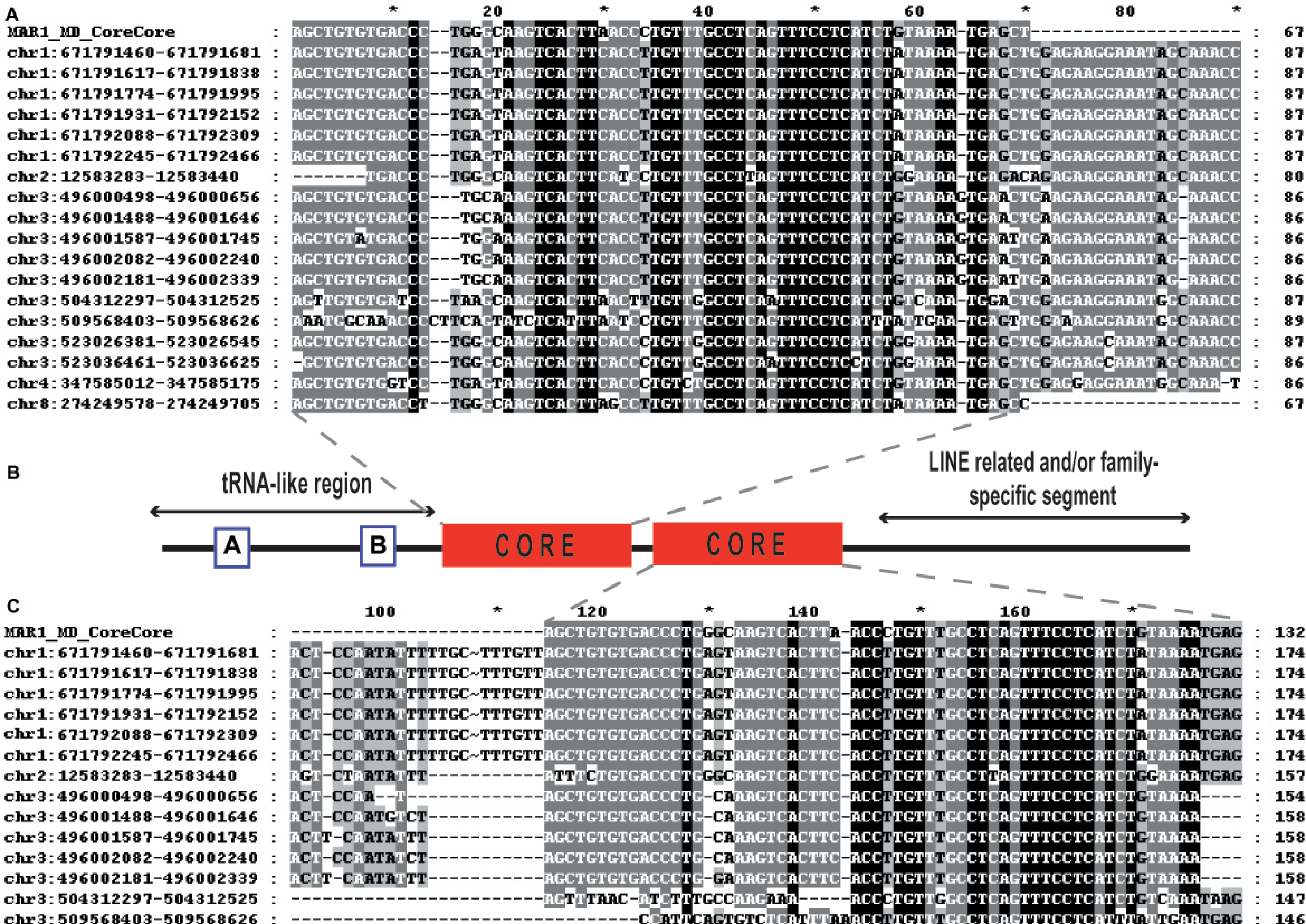
A
MAR1_MD_CoreCore
B
tRNA-like region
A
B
CORE
CORE
LINE related and/or family-specific segment
C
MAR1_MD_CoreCore
