# Supplementary material for: Ancient Exaptation of a CORE-SINE Retroposon into a Highly Conserved Mammalian Neuronal Enhancer of the Proopiomelanocortin Gene
Source: PLoS Genet. 2007 Oct 5;3(10):e166. doi: 10.1371/journal.pgen.0030166 (PMC2000970; doi:10.1371/journal.pgen.0030166)
Supplement: Supplementary file 5 [file pgen.0030166.sg005.pdf]

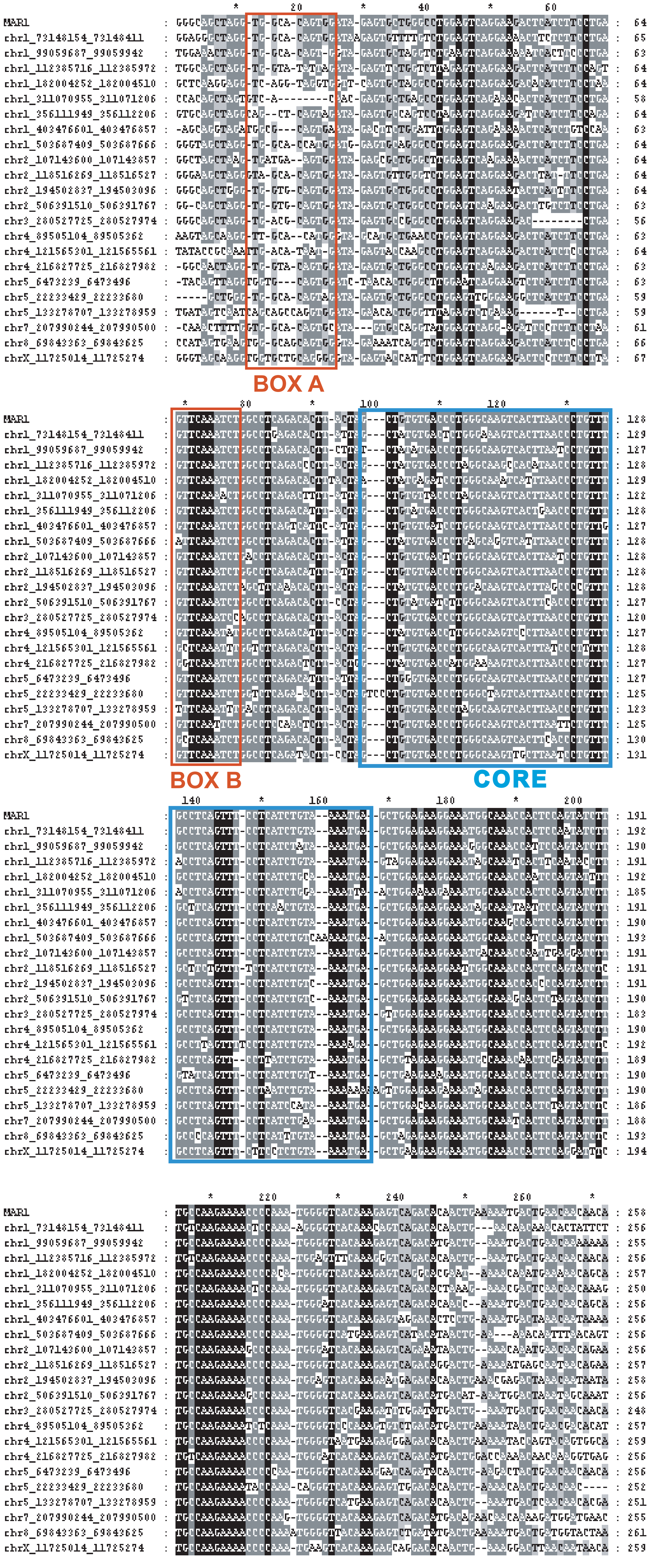
BOX A
BOX B
CORE
MAR1
chr1_73148154_73148411
chr1_99059687_99059942
chr1_112385716_112385972
chr1_182004252_182004510
chr1_311070955_311071206
chr1_356111949_356112206
chr1_403476601_403476857
chr1_503687409_503687666
chr2_107143600_107143857
chr2_118516269_118516527
chr2_194502837_194503096
chr2_506391510_506391767
chr3_280527725_280527974
chr4_89505104_89505362
chr4_121565301_121565561
chr4_216827725_216827982
chr5_6473239_6473496
chr5_22233429_22233680
chr5_133278707_133278959
chr7_207990244_207990500
chr8_69843363_69843625
chrX_11725014_11725274
