# Supplementary material for: Ancient Exaptation of a CORE-SINE Retroposon into a Highly Conserved Mammalian Neuronal Enhancer of the Proopiomelanocortin Gene
Source: PLoS Genet. 2007 Oct 5;3(10):e166. doi: 10.1371/journal.pgen.0030166 (PMC2000970; doi:10.1371/journal.pgen.0030166)
Supplement: Supplementary file 6 [file pgen.0030166.st001.doc]

**Table S1:** Summary of the expression analysis performed by X-Gal/ACTH double histochemistry on adult brains at the arcuate level of WT nPE2 and the 5 deletion constructs**.**

| Name of transgene | Number of transgenic lines | Number of transgenic lines showing X-Gal/ACTH colocalization in POMC arcuate neurons | Lines with ectopic staining only | Lines with no staining |
| --- | --- | --- | --- | --- |
| nPE2-lacZ | 5 | 3 | 0 | 2 |
| Δ1nPE2-lacZ | 5 | 0 | 1 | 4 |
| Δ2nPE2-lacZ | 5 | 2 | 1 | 2 |
| Δ3nPE2-lacZ | 6 | 0 | 1 | 5 |
| Δ4nPE2-lacZ | 5 | 3 | 0 | 2 |
| Δ5nPE2-lacZ | 12 | 8 | 0 | 4 |
