# Supplementary material for: Ancient Exaptation of a CORE-SINE Retroposon into a Highly Conserved Mammalian Neuronal Enhancer of the Proopiomelanocortin Gene
Source: PLoS Genet. 2007 Oct 5;3(10):e166. doi: 10.1371/journal.pgen.0030166 (PMC2000970; doi:10.1371/journal.pgen.0030166)
Supplement: Supplementary file 7 [file pgen.0030166.st002.doc]

**Table S2.** Identity values (% identical nucleotides) among consensus MAR1 MD and the top 20 most similar sequences retrieved after Blast searches at the *Monodelphis domestica* genome. Genomic location of the instances is given according to the Genome Browser at UCSC.

|  | **REGIONS** | | |
| --- | --- | --- | --- |
| **MAR1 MD** | **tRNA-like** | **CORE** | **LINE related and/or family-specific segment** |
| **chr1:73148154_73148411** | **86** | **95** | **79** |
| **chr1:99059687_99059687** | **82** | **93** | **91** |
| **chr1:182004252_182004510** | **87** | **91** | **86** |
| **chr1:311070955_311071206** | **78** | **90** | **86** |
| **chr1:356111949_356112206** | **74** | **91** | **86** |
| **chr1:403476601_403476857** | **86** | **93** | **91** |
| **chr1:503687409_503687666** | **73** | **96** | **90** |
| **chr2:107143600_107143857** | **92** | **89** | **78** |
| **chr2:118516269_118516527** | **87** | **96** | **83** |
| **chr2: 194502837_194503096** | **90** | **95** | **85** |
| **chr2:506391510_506391767** | **91** | **91** | **89** |
| **chr3:280527725_280527974** | **92** | **90** | **85** |
| **chr4:89505104_89505362** | **86** | **100** | **90** |
| **chr4:121565301_121565561** | **83** | **95** | **88** |
| **chr4:121565301_121565561** | **75** | **92** | **85** |
| **chr4:216827725_216827982** | **92** | **90** | **76** |
| **chr5:6473239_6473496** | **80** | **93** | **89** |
| **chr5:22233429_22233680** | **87** | **93** | **83** |
| **chr5:133278707_133278959** | **62** | **95** | **90** |
| **chr7:207990244_207990500** | **74** | **95** | **83** |
| **chr8:69843363_69843625** | **78** | **95** | **83** |
| **chrX:11725014_11725274** | **81** | **91** | **85** |
